# Reliability and validity of the AGREE instrument used by physical therapists in assessment of clinical practice guidelines

**DOI:** 10.1186/1472-6963-5-18

**Published:** 2005-03-02

**Authors:** Joy C MacDermid, Dina Brooks, Sherra Solway, Sharon Switzer-McIntyre, Lucie Brosseau, Ian D Graham

**Affiliations:** 1 School of Rehabilitation Science, McMaster University, Hamilton, Ontario, Canada; 2 Upper Limb Centre Clinical Research Laboratory, St. Joseph's Health Centre, 268 Grosvenor St., London, Ontario, N6A 3A8, Canada; 3 Department of Physical Therapy, University of Toronto, Toronto, Canada; 4 Division of Clinical Decision-Making and Health Care, University Health Network, Toronto, Canada; 5 School of Rehabilitation Sciences, Faculty of Health Sciences, University of Ottawa, Canada; 6 School of Nursing, University of Ottawa, Ottawa, Canada; 7 Ottawa Health Research Institute, Ottawa, Canada

## Abstract

**Background:**

The AGREE instrument has been validated for evaluating Clinical Practice Guidelines (CPG) pertaining to medical care. This study evaluated the reliability and validity of physical therapists using the AGREE to assess quality of CPGs relevant to physical therapy practice.

**Methods:**

A total of 69 physical therapists participated and were classified as generalists, specialist or researchers. Pairs of appraisers within each category evaluated independently, a set of 6 CPG selected at random from a pool of 55 CPGs.

**Results:**

Reliability between pairs of appraisers indicated low to high reliability depending on the domain and number of appraisers (0.17–0.81 for single appraiser; 0.30–0.96 when score averaged across a pair of appraisers). The highest reliability was achieved for Rigour of Development, which exceeded ICC> 0.79, if scores from pairs of appraisers were pooled. Adding more than 3 appraisers did not consistently improve reliability. Appraiser type did not determine reliability scores. End-users, including study participants and a separate sample of 102 physical therapy students, found the AGREE useful to guide critical appraisal. The construct validity of the AGREE was supported in that expected differences on Rigour of Development domains were observed between expert panels versus those with no/uncertain expertise (differences of 10–21% p = 0.09–0.001). Factor analysis with varimax rotation, produced a 4-factor solution that was similar, although not in exact agreement with the AGREE Domains. Validity was also supported by the correlation observed (Kendall-tao = 0.69) between Overall Assessment and the Rigour of Development domain.

**Conclusion:**

These findings suggest that the AGREE instrument is reliable and valid when used by physiotherapists to assess the quality of CPG pertaining to physical therapy health services.

## Background

Clinical practice guidelines (CPGs) are one option for promotion of quality in health services [1-6] Many countries are faced with common challenges in delivering high-quality health care with available resources and have pursued the development of CPGs as a means to optimize effective and efficient care. As a result there is a need to evaluate CPGs guidelines to assess their quality and their impact on practice. The Appraisal of Guidelines for Research and Evaluation (AGREE) instrument was developed by a group of researchers from 13 countries to provide a systematic framework for assessing guideline quality[7,8] This instrument was thoroughly evaluated and refined and is now a commonly used assessment instrument for CPGs[2,3,9-13] A large-scale validation study focussing primarily on medical (i.e. physician) guidelines, was conducted supporting the reliability and validity of this instrument[14].

The AGREE Collaboration published the development process and associated reliability and validity data in 2003[14] This report outlined the rigorous process undertaken to develop the AGREE instrument which included item generation, selection and scaling followed by field-testing and refinement. This process resulted in the final instrument with 23 items distributed across six subscales termed "domains", for which reliability and validity data were presented. Reliability was determined by calculating the internal consistency of each domain within the final instrument and assessing the agreement between different appraisers. A total of 33 guidelines were evaluated by the four appraisers. Internal consistency ranged from 0.64–0.88. 'Scope of Purpose' and 'Rigour of Development' were the most homogeneous domains. The inter-rater reliability exhibited a wide range from 0.25–0.91. Reliability was higher with four appraisers and the most reliable domain was 'Rigor of Development'. Higher reliability within the domain of 'Rigor of Development' is a positive finding, as this domain should contain items that are more objective than items contained on other subscales of the AGREE instrument. That is, because the 'Rigor of Development' questions relate to the methodology of developing a CPG and thus there are optimal criteria that would be expected regardless of the content of the CPG. In measurement terms, it is more likely that a "true" score exists for elements within this domain. Therefore, variability observed on repeated assessments of the same CPG should reflect measurement error between appraisers. Other scales such as stakeholder involvement and applicability might reasonably have different criteria depending on clinical expertise or application. In measurement terms, no single true score may exist for these items. Therefore, variability observed between appraisers on these domains might reflect a combination of measurement error, as well as true variations in perspective. This concept is important when assessing and interpreting the reliability of evaluation instruments like the AGREE.

The AGREE Collaboration also assessed face, construct and criterion validity of the AGREE Instrument. Face validity was determined by surveying appraisers attitudes and opinions about the instrument and its associated user guide. Construct validity was determined by comparing scores of guidelines in different subgroups to determine whether they fit three specific constructs. The constructs tested included whether established quality guideline programs produced guidelines with higher domain scores than those developed outside of established systems; whether guidelines supported by well-documented technical information had higher domain scores than those without such documentation; and finally whether guidelines developed as national policies were higher quality than regional or local CPGs. The first hypothesis was supported with respect to editorial independence, but not other domains. The second and third hypotheses were supported with respect to the domain Rigor of Development, but not other domains. Finally, criterion validity was determined by assessing the rank correlation between appraisers domain scores and their overall assessment scores (final item on the AGREE instrument). These correlations were all highly significant (range Kendall's Tau-b = 0.67 – 0.88).

Physical therapy and other health care disciplines shares common challenges in providing effective care within limited resources. While many disciplines currently use the AGREE the validation paper emphasized medical practice and practitioners. The nature of physical therapy practice differs substantively from medical practice in a number of ways including access, funding, the nature of interventions, research systems and professional associations; all of these might affect the type of CPGs, developed. Differences in training between disciplines might also cause variations in how the AGREE was interpreted. Additional validation with other settings or users would strengthen the validation of the AGREE across a variety of applications.

CPGs have arisen within the field of physical therapy from a variety of sources[15,16] Professional associations have assisted physical therapy practitioners in becoming aware of the existence of such guidelines through websites and newsletters. Ideally, information on the quality of such guidelines should also be available to assist practitioners in selecting between available guidelines. Currently, this is not the case in physical therapy. While professional associations may help co-ordinate the evaluation of CPGs, they must inevitably must make decisions about this process including which members of the profession are able to evaluate CPGs, how many appraisers should be selected and which instrument should be used. For this reason, our purpose was to determine whether the AGREE instrument is a reliable and valid tool when used by physical therapists to assess CPGs that pertain to physical therapy practice. Our hypothesis was that the AGREE instrument would be a valid tool when used by physical therapists to evaluate CPGs. Our secondary question was whether an ideal number or type of physical therapist appraiser would be evident from reliability data. We hypothesized that 4 appraisers might be best, as the AGREE Collaboration recommends " al least two appraisers and preferably four as this will increase the reliability of the assessment". [7]

## Methods

This study was a cross-sectional study conducted to evaluate inter-appraiser reliability and validity of quality appraisal of CPGs performed by physical therapists using the AGREE instrument Permission to use the AGREE instrument was obtained from the AGREE Collaboration. Ethics approval for this study was received from the University of Toronto, Office of Research Services.

### Clinical practice guidelines

Clinical practice guidelines (CPGs) are "systematically developed statements to assist practitioner and patient decisions about appropriate healthcare for specific clinical circumstances"[17]. The CPGs evaluated during this study were identified through an inventory that was created by the study authors from a series of systematic searches that included electronic databases, websites, contact of professional associations and guideline developers. The inventory included all located documents that were identified by authors as Clinical Practice Guidelines. This inventory was completed in 2002 and updated yearly and subsequently posted on the website for the Canadian and Ontario Physiotherapy Associations (members only access). Within this database, guidelines were categorized according to the area of physical therapy practice (e.g., musculoskeletal, neurological, cardiorespiratory). Sixty guidelines published in the last five years were selected from this database for inclusion in the present study. Four of these CPGs were excluded because they were not actually CPGs (e.g. systematic reviews included in error) and one CPG was excluded because it was not relevant to physical therapy. Thus in total, a sample of fifty-five guidelines were evaluated in the present study.

### Participants/training

All participants were physical therapists who were recruited through advertisements in professional newsletters. A total of 72 therapists responded to advertisements and agreed to participate, 69 actually participated in training and study evaluations – two others had personal circumstances that prevented them from attending training and one failed to respond further. The participants were classified according to the following criteria: 1. Clinical Specialists were Physical Therapists who were currently practicing in a specific area, had a minimum of three years experience and had participated in at least one post-graduate course per year in their area of clinical expertise. 2. Generalists were Physical Therapists practicing in a variety of areas of physical therapy or an ongoing general practice, which covered a broad-spectrum of neurological, orthopaedic and cardiorespiratory health problems. 3. Researchers were Physical Therapists with or approaching completion of a graduate research degree (M.Sc. and/or PhD) with experience in conducting clinical trials or outcomes research and experience in formal critical appraisal of clinical research.

Demographic data was collected on all participants. Eligible participants were provided with training materials which included the AGREE instrument (form and associated interpretation guidelines)[7,18] as well as a multiple-choice test that required participants to answer questions on the content and structure of the AGREE (see Additional file 1). In addition, a sample guideline was provided to participants with instructions to read the guideline (on management of lymphedema following treatment for breast cancer[19]) and appraise it using the AGREE instrument and associated documentation.

Subsequently, all participants met by teleconference for one hour with a subgroup (4–8) of study participants. The sessions (a total of 6 teleconferences conducted) were led by a single facilitator (first author). During this session participants reviewed their responses to each question and discussed their rationale or concerns regarding scoring. The facilitator did not indicate a correct score for any individual item. Participants were instructed that the group facilitator would not indicate whether a given score was correct, as this was not possible for many of the items. Participants were directed to appreciate the difference between items that had clear answers because they inquired about specific factual information versus those that had a subjective element where responses might vary according to context. Participants were corrected if they incorrectly interpreted the meaning of a given item on the AGREE instrument. Although consensus was not the objective of the training sessions, participants tended to come to consensus after discussing items with colleagues and a facilitator. In total, 69 appraisers attended the training sessions.

### Appraisal instrument

The appraisal instrument used to evaluate the CPGs was the AGREE [18]. This instrument consists of 23 items organized in six domains ; each domain is intended to capture a separate dimension of guideline quality. The following domains are included:

1. *Scope And Purpose: *3 items that address the overall aim of the guideline, the clinical question and the target population

2. *Stakeholder Involvement: *4 items that address the composition, expertise and representation of the development group

3. *Rigor Of Development: *7 items that evaluate the process used to locate and synthesize the evidence and to formulate and update the recommendations,

4. *Clarity And Presentation: *4 items that address language and format

5. *Applicability: *3 items that address the potential organizational, behavioural and cost implications of implementation and

6. *Editorial Independence: *2 items that address potential conflicts of interest.

Items are rated on a 4-point scale with endpoints of 4 'strongly agree' and 1 'strongly disagree'; the two midpoints are 3 'agree' and 2 'disagree'. A section for overall assessment is included at the end of the instrument that requires the appraiser to make a judgment about the overall quality of the CPG. Appraisers are asked whether they would 'strongly recommend', 'recommend (with provisos or alterations)', 'would not recommend' or are 'unsure' if they would recommend the CPG for practice.

### Evaluation process

All participants completed the training program and proceeded to evaluate a set of 6 CPGs. These CPGs, six copies of the AGREE instrument, and a pre-paid return were provided by mail. Each guideline was evaluated independently by three pairs of appraisers who were randomly picked from the three pools of Physical Therapists (i.e. two clinical specialists in the area of the CPG, two generalists and two researchers). All appraisers returned their packages, although up to 3 reminders calls were required for late returns. Participants who completed the study were provided with an honorarium ($100).

### Data analysis

Data analysis was conducted to verify the quality of data, assess instrument reliability and determine the validity of the AGREE instrument for physical therapy practice. SPSS statistical software for Windows (Version 11.0; SPSS Inc, Chicago, Illinois) was used for all statistical analyses. P-values of 0.05 or less were considered significant.

### Data Entry/Quality

Data entry was completed by a single research assistant who inspected data for errors once the data file was complete. The first author conducted random checks of data entry against original data sheets. Descriptive statistics were conducted to identify outliers or unusual values.

Domain scores of each CPG were calculated as recommended by the AGREE Collaboration. The scores of the individual items in the domain were summed and standardized as a percentage of the maximum possible score for that domain (AGREE Collaboration, 2001).



### Reliability analyses

The internal consistency of each domain was evaluated using Cronbach's Alpha. The reliability between appraisers was determined for each question and each domain of the AGREE. Intraclass correlation coefficients (ICC 1,1) were calculated within each pair of appraisers and across the pool of appraisers. A one-way random effects model was used as pairs of appraisers were randomly selected from our pool of physical therapist appraisers. An unweighted and quadratic weighted kappa were calculated to indicate the agreement within pairs of appraisers on whether a CPG was appropriate for clinical utilization.

ICCs or kappa values above 0.75 were considered to represent good, 0.40–0.75 moderate and <0.40 poor reliability[20]

### Validity analyses

#### Face validity

The face validity of the instrument for physical therapy practice was determined from feedback provided on the instrument from two sources. Participants (experienced physical therapists) in the study were invited to provide feedback at the training sessions (open-ended questions regarding the training session and the AGREE itself -verbal response). They were also asked to add comments about any items, any issues with clarity or concerns directly on their AGREE form when they were using the AGREE on their assigned CPGs. These were returned, by mail, with their ratings. In addition, over the course of two years, a sample of 102 entry-level-masters trainees at McMaster University were provided the training materials (except for multiple-choice questionnaire) and were required to complete an assignment where they evaluated the same CPG[19] used during the study training session. This assignment consisted of a facilitated group component where students worked in groups of 4–6 to complete the AGREE evaluation for the assigned CPG. The individual component of this assignment required each student to write a 1–2 page essay evaluating the instrument itself in terms of its relevance to clinical practice, validity and their personal preference about whether they would use it again. This information was summarized by the course instructor (first author) and the percentage of students who responded that they would use the instrument again was tabulated.

#### Factor (domain) validity

The validity of the domain structure was evaluated using a principal components, varimax rotated factor analysis. Item means across all 6 appraisers were entered into the analysis. Coefficients were evaluated to determine whether they supported the domain structure and followed a similar pattern as to that reported for a previous factor analysis published by the AGREE Collaboration.

#### Construct Validity

Construct validity was assessed by evaluating 2 hypotheses. The first hypothesis was selected to match the hypothesis tested by the AGREE Collaboration guidelines[14] and supported by others[21,22], that CPGs developed by established guideline developers should have higher quality scores than those created outside of established system. All guidelines in the database were classified as having been developed by established guideline developers if it could be identified that an experienced guideline developer or development group was responsible for a specific guideline. A CPG was classified as having "Experienced Guideline Developers" if it 1) had more than 3 authors (also fulfilled by an agency) and 2) at least one team member could be identified an a methodologist experienced in CPG development – either by descriptions contained within the body or the CPG or after a review of listed authors (conducted by JM and DB). If this could not be verified the CPG was classified as " No or Uncertain Methodology Expertise". Our second hypothesis was that physical therapists would be more likely to recommend a guideline that was rated as having higher Rigor of Development scores. An independent t-test was used to evaluate the scores obtained for Rigor of Development for guidelines judged as acceptable versus those that were not. This hypothesis test is not ideal, as we are testing whether this subscale contributes to the overall rating within the same instrument. Nevertheless, in the absence of an external criterion, we choose to use this analysis given that it was also conducted in the original validation paper and there was an advantage to having a comparable analysis.

#### Criterion validity

Finally, criterion validity was assessed in the same manner as reported by the AGREE Collaboration in their validation study. Again we recognize we did not have an external criterion. Kendall Tau B Rank correlation coefficients were calculated between the appraisers domain scores and the overall assessment score.

## Results

### Participants

Sixty-nine physical therapists were recruited and were categorized as clinical specialists (n = 29), generalists (n = 21) or researchers (n = 19) (Table 1). Generalists and specialists reported similar years of clinical experience with generalists ranging from 3–33 (mean ± SD,16 ± 11) years and specialists 3–35 (15 ± 8) years. Researchers reported 3–21 (7 ± 6) years of research experience.

**Table 1:** Demographic description of physical therapy evaluators

**Group**	**Age Mean (SD)**	**Gender %**		**Highest Degree %**			
		**Female**	**Male**	**Diploma**	**Bachelors**	**Masters**	**Doctorate**

**Overall **(n = 69)	40 (8)	96	4	9	58	32	1
**Generalists **(n = 21)	39 (9)	86	14	19	71	10	0
**Specialists **(n = 29)	39 (7)	100	0	7	79	14	0
**Researchers **(n = 19)	41 (9)	100	0	0	11*	84	5

* these two participants had significant research experience and were close to completion of Masters degree

The majority of specialists were orthopaedic physical therapists (55%), followed by neurological (24%) and cardiorespiratory (10%). One participant reported to specialize in paediatric physical therapy. Two participants did not specify their area of specialization.

### Reliability

Analysis of reliability of individual items indicates a trend for higher reliability in items within the domain from Rigor of Development (See Table 2). Intraclass correlation coefficients (ICCs) for each domain of the AGREE instrument for pairs of appraisers are presented in Table 3. No systematic differences were observed that would indicate that type of appraisers had any substantial impact on the reliability obtained. Variation in reliability was observed across domains with Rigor of Development demonstrating the highest level of reliability. Few ICCs reached the excellent benchmark of 0.75, if a single appraiser performed the evaluation. ICC models that estimate the reliability when appraisers were averaged using models (1,2) for pairs or (1,6) across all six appraisers indicated substantial improvement in reliability if appraisals were averaged across appraisers. When comparing the reliability across different numbers of appraisers (Table 4) the improvement in reliability was most notable when going from two to three appraisers, with the exception of editorial independence). Additional benefit for adding additional appraisers was inconsistent. Agreement on the overall assessment of the CPG had low reliability for generalists and specialists and moderate reliability for researchers. Quadratic weighting demonstrated some improvement in reliability coefficients for generalists and researchers, but not for specialists. (Table 5)

**Table 2:** Inter-rater reliability of AGREE instrument – individual items

	**Generalists**		**Clinical Specialists**		**Researchers**		**Overall**	
	
	**Single Rater ICC**	**Average of Raters ICC**	**Single Rater ICC**	**Average of Raters ICC**	**Single Rater ICC**	**Average of Raters ICC**	**Single Rater ICC**	**Average of Raters ICC**
**Item 1**	0.34	0.51	0.50	0.66	0.58	0.73	0.46	0.84
**Item 2**	0.28	0.44	**0.03**	**0.05**	**-0.09**	**-0.19**	**0.25**	**0.67**
**Item 3**	0.45	0.62	0.23	0.37	**0.19**	**0.31**	0.22	0.63
**Item 4**	0.58	0.74	0.65	0.79	0.75	0.86	0.67	0.92
**Item 5**	0.57	0.73	0.37	0.54	0.55	0.71	0.45	0.83
**Item 6**	0.43	0.60	0.62	0.76	0.69	0.82	0.55	0.88
**Item 7**	0.48	0.65	**0.18**	**0.30**	0.36	0.53	0.36	0.77
**Item 8**	0.88	0.93	0.77	0.87	0.78	0.88	0.83	0.97
**Item 9**	0.72	0.84	0.84	0.91	0.69	0.82	0.72	0.94
**Item 10**	0.61	0.76	0.54	0.70	0.66	0.80	0.62	0.91
**Item 11**	0.45	0.63	**0.13**	**0.23**	0.32	0.49	0.39	0.79
**Item 12**	0.61	0.75	0.46	0.63	0.55	0.71	0.63	0.91
**Item 13**	0.74	0.85	0.66	0.79	0.56	0.72	0.64	0.92
**Item 14**	0.57	0.73	0.61	0.76	0.70	0.82	0.65	0.92
**Item 15**	0.31	0.47	0.30	0.46	**0.25**	**0.40**	0.38	0.79
**Item 16**	0.39	0.56	0.49	0.66	0.46	0.63	0.43	0.82
**Item 17**	0.31	0.48	0.31	0.48	0.41	0.58	0.35	0.76
**Item 18**	0.32	0.48	0.60	0.75	0.42	0.59	0.52	0.87
**Item 19**	0.53	0.69	0.50	0.67	0.45	0.62	0.43	0.82
**Item 20**	0.48	0.65	0.40	0.57	0.49	0.66	0.43	0.82
**Item 21**	0.34	0.51	0.37	0.54	**0.20**	**0.34**	0.28	0.70
**Item 22**	0.53	0.69	**0.15**	**0.27**	0.42	0.60	0.26	0.68
**Item 23**	0.58	0.73	0.53	0.69	0.72	0.84	0.40	0.80

ICC – Intraclass correlation coefficient

Results statistically significant at the p < 0.05 level except where indicated by **bold.**

**Table 3:** Inter-rater reliability of AGREE instrument domain scores

	**Scope & Purpose**		**Stakeholder Involvement**		**Rigor of Development**		**Clarity & Presentation**		**Applicability**		**Editorial Independence**	
	
	**Single Rater ICC *(95% CI)***	**Average of Raters ICC- all 6 raters or 3 pairs of 2 raters? *(95% CI)***	**Single Rater ICC *(95% CI)***	**Average of Raters ICC *(95% CI)***	**Single Rater ICC *(95% CI)***	**Average of Raters ICC *(95% CI)***	**Single Rater ICC *(95% CI)***	**Average of Raters ICC *(95% CI)***	**Single Rater ICC *(95% CI)***	**Average of Raters ICC *(95% CI)***	**Single Rater ICC *(95% CI)***	**Average of Raters ICC *(95% CI)***
**Generalists**	0.37 (0.11–0.59)	0.54 (0.19–0.74)	0.71 (0.54–0.83)	0.83 (0.70–0.90)	0.81 (0.68–0.89)	0.89 (0.81–0.94)	0.41 (0.15–0.62)	0.58 (0.26–0.76)	0.65 (0.46–0.79)	0.79 (0.63–0.88)	0.60 (0.39–0.75)	0.75 (0.56–0.86)
**Clinical Specialists**	0.35 (0.10–0.56)	0.52 (0.18–0.72)	0.59 (0.39–0.74)	0.74 (0.56–0.85)	0.65 (0.47–0.78)	0.79 (0.64–0.88)	0.51 (0.29–0.69)	0.68 (0.45–0.81)	0.43 (0.19–0.63)	0.61 (0.32–0.77)	0.32 (0.06–0.54)	0.49 (0.11–0.70)
**Researchers**	0.17 (0.14–0.46)	**0.30 **(-0.32–0.63)	0.73 (0.54–0.85)	0.84 (0.70–0.92)	0.77 (0.61–0.87)	0.87 (0.76–0.93)	0.47 (0.18–0.69)	0.64 (0.31–0.81)	0.47 (0.19–0.68)	0.64 (0.32–0.81)	0.59 (0.34–0.77)	0.75 (0.51–0.87)
**Overall**	0.40 (0.25–0.58)	0.80 (0.66–0.89)	0.67 (0.54–0.80)	0.93 (0.87–0.96)	0.79 (0.68–0.88)	0.96 (0.93–0.98)	0.55 (0.40–0.72)	0.88 (0.80–0.94)	0.50 (0.35–0.68)	0.86 (0.76–0.93)	0.35 (0.19–0.54)	0.76 (0.59–0.88)

CI – confidence interval; ICC – intraclass correlation coefficient

Results statistically significant at the p < 0.5 level except where indicated by **bold.**

**Table 4:** Intraclass correlations for each AGREE instrument domain as a function of the number of raters

	**Scope & Purpose**		**Stakeholder Involvement**		**Rigor of Development**		**Clarity & Presentation**		**Applicability**		**Editorial Independence**	
	
	**Single Rater ICC**	**Average of Raters ICC**	**Single Rater ICC**	**Average of Raters ICC**	**Single Rater ICC**	**Average of Raters ICC**	**Single Rater ICC**	**Average of Raters ICC**	**Single Rater ICC**	**Average of Raters ICC**	**Single Rater ICC**	**Average of Raters ICC**
**2 Raters**	0.37	0.54	0.71	0.83	0.81	0.89	0.41	0.58	0.65	0.79	0.60	0.75
**3 Raters**	0.38	0.64	0.71	0.88	0.82	0.93	0.54	0.78	0.66	0.85	0.38	0.65
**4 Raters**	0.41	0.73	0.64	0.88	0.77	0.93	0.51	0.80	0.54	0.82	0.35	0.69
**5 Raters**	0.42	0.78	0.69	0.92	0.79	0.95	0.51	0.84	0.57	0.87	0.38	0.76
**6 Raters**	0.40	0.80	0.67	0.93	0.79	0.96	0.55	0.88	0.51	0.86	0.35	0.76

ICC = Intraclass correlation coefficient

**Table 5:** Agreement on whether a CPG would be recommended or not

Pair of Raters	Kappa (unweighted)	Kappa (quadratic weights)
Generalists	0.20	0.34
Specialists	0.25	0.22
Researchers	0.39	0.47

A Kappa was calculated on the final overall rating question whether or not a CPG should be using with the data dichotomized as YES (strongly recommend or recommend with provisos) or NO (Would not recommend or unsure) or by using quadratic weighting to compare the strength of recommendation (Strongly, with provisos, would not, unsure).

### Validity

#### Face Validity/ User Feedback

Study participants provided feedback during training sessions primarily with respect to the training session itself. They found the opportunity to discuss the results with others to be useful as a means of understanding the intent of individual items. Only three participants had previously been exposed to the AGREE instrument and that majority expressed positive comments about the value of learning about the AGREE instrument. Some clinicians expressed some anxiety about the role for CPGs and how they might be used. None of the study participants provided any feedback when returning their mail packages.

The entry-level physical therapists (students) uniformly agreed that the AGREE instrument provided a useful structure and guidance in the evaluation of the CPG. Students compared the AGREE instrument to evaluation instruments they had used for critical appraisal of different study designs, such as clinical trials and systematic reviews. Students frequently commented that these previous instruments had a more open-ended format and expected the reviewer to understand issues in critical appraisal with little direction as to expectations or scoring criteria. Thus, they found the concrete nature of the AGREE instrument and the clear instruction on interpretation to be a useful framework for the evaluation process. Students stated that this direction increased their confidence that they had addressed all important issues. Although students differed on their ratings for individual questions, as well as the overall usefulness of the CPG evaluated, the majority of students understood and correctly interpreted the intent of the items from all of the AGREE domains. The majority of the students, 96 %, stated that they would use the AGREE instrument in other situations. A concern raised by the remaining 4% and other students who would continue to use the instrument was the length of the form and the amount of time required to complete the evaluation, given the busy nature of clinical practice.

#### Factor analysis

The factor analysis supported a 4-factor solution. The first factor explained 45% of the variance and contained items primarily from the Scope and Purpose or Rigor of Development domains. The second component explained 12% of the variance and contained items primarily from the Clarity and Presentation or Applicability domains. The third factor explained 7.7% of the variance and contained all of the items from the Stakeholder Involvement domain, all of the Editorial Independence items and question 13 from Rigor of Development, which pertains to whether the guideline has been externally reviewed by experts. The fourth component explained 5.6 percent of the variance and contained item 11 regarding health benefits/side effects and item 15 regarding whether recommendations were specific and unambiguous. Item means and their loadings are presented in Table 6.

**Table 6:** Results of factor analysis (principal components with varimax rotation)

	Item Mean	Std. Deviation	Components			
			1	2	3	4

Scope and Purpose						

Q1	3.4003	.72614	**.690**	.339	.271	.217
Q2	2.9681	.68948	**.803**	.213	.129	.296
Q3	3.3961	.51941	.393	**.489**	.016	.430
Stakeholder Involvement						
Q4	2.6814	1.07262	.336	.422	**.661**	.093
Q5	1.7517	.89553	.179	.173	**.739**	.058
Q6	2.8331	.89717	.195	.294	**.726**	.259
Q7	1.6664	.75710	.255	.290	**.675**	.175
Rigour of development						
Q8	2.7344	1.21287	**.902**	.096	.247	-.087
Q9	2.6533	1.20139	**.913**	.112	.229	-.123
Q10	2.6314	1.05247	**.800**	.132	.384	.066
Q11	2.9492	.76769	.059	.339	.302	**.682**
Q12	2.9811	1.00409	**.626**	-.058	.223	.514
Q13	2.3556	1.16306	.422	.273	**.617**	.213
Q14	1.9425	.99370	.323	**.668**	.090	.044
Clarity and presentation						
Q15	3.2250	.73262	.132	.200	.149	**.864**
Q16	3.2269	.77070	.022	**.607**	.241	.322
Q17	3.1631	.71880	.093	**.606**	.453	.400
Q18	2.3342	.98180	.091	**.671**	.449	.273
Applicability						
Q19	2.2072	.94797	.158	**.830**	.231	.005
Q20	1.9500	.83211	.172	**.818**	.176	-.073
Q21	2.2336	.80322	-.017	**.775**	.299	.237
Editorial Independence						
Q22	2.1453	.90158	.451	.108	**.669**	.045
Q23	1.7575	.92802	.178	.305	**.452**	-.436

This table presents the results of the final 4 factor solution to factor analysis. **Bolded **cells shown the factor for which each item loaded most strongly. Results are grouped according to the Domains of the AGREE.

#### Construct validity

The construct that the CPGs developed by expert guideline development groups would have a higher score on the domain Rigor of Development was supported (See Table 7). The construct that therapists would be more likely to recommend for usage a CPG with a higher quality on the domain Rigor of Development was also supported (mean of 76 vs. 58 p <0.001).

**Table 7:** Hypothesis test: rigour of development is greater where panel is known to have methodology expertise

Rater	Expert Panel	No or Uncertain Expertise	p
Generalist #1	79	58	0.002
Generalist #2	79	57	0.001
Specialist #1	73	58	0.015
Specialist #2	75	65	0.09
Researcher #1	72	57	0.02
Research #2	80	61	0.014
All 6 appraisers combined	78	59	<0.001

This table contains the scores for the Agree Domain on Rigour of Development. CPGs were classified as having an expert panel if 1) there were more than 3 authors listed (or an agency) and 2) there was an experienced CPG methodologist clearly identified as a panel member or if one of the study investigators was recognized as such. All others were classified as "No or Uncertain Expertise". The p value for the independent samples t-test is shown.

#### Criterion validity

The correlation between overall assessment and the domain scores ranged from low (0.38) to moderate (0.64), with the highest correlation being observed for the Rigor Of Development domain. (Table 8)

**Table 8:** Correlation of domain scores with overall assessment of AGREE

Domain	Correlation with Overall Rating
Stakeholder Involvement	0.59
Scope and Purpose	0.52
Rigour of Development	0.64
Clarity and Presentation	0.62
Applicability	0.49
Editorial Independence	0.38

Kendall's Tau-b correlations were conducted between the mean rating of the over assessment of the CPG across all raters as compared to the mean of each Domain score. As hypothesized the correlation was highest with Rigour of development.

## Discussion

The study findings suggest that the AGREE instrument is reliable and valid when used by physical therapists to evaluate CPGs. While some differences exist between the results reported in the original validation study authored by the AGREE Collaboration, the similarities far outweigh the differences. This would suggest that the process of evaluating CPGs using the AGREE instrument can be transferred to physical therapy practice to support the translation of higher quality CPGs into physical therapy health services.

Typically, individual item reliability is of little relevance when evaluating the properties of a measurement scale, as items are not intended to be used in isolation. However, it may be useful from a practical point of view to form hypotheses about were further training might be necessary, if it appears that appraisers have particular difficulty with certain items. As discussed above, one must be careful in interpreting reliability coefficients in isolation, particularly for instruments like the AGREE where some items have "relatively true" scores and others have a spectrum of true scores. For the AGREE this spectrum might exist on application items. Thus, we would expect low item reliability might result from items where there is large measurement error, but also from items where there is substantial variability in how the item might apply in different circumstances. Our data support this. Low reliability was evident on items such as whether the guideline was editorially independent (2/8 ICCs were not greater than 0) and whether key review criteria for monitoring or audit had been provided (2/8 ICCs were not greater than 0). Study participants also appeared to have difficulty interpreting these items during the training session and thus these disagreements may reflect a lack of consensus about the meaning of these items. In some CPGs information on Editorial Independence is contained in footnotes and was missed by some evaluators. Improvements in reliability on these items might be anticipated with further training. Other items where low reliability was observed included whether the clinical question was specifically described (6/8 ICCs were not greater than 0) and whether the patients to whom the guideline applied were specifically described (2/8 ICCs were not greater than 0). During training, participants appeared to understand these questions, although expectations about what constituted an appropriate description of the clinical question or patient population tended to vary according to the participant's level of expertise in that given clinical area. Thus, disagreements on these items may partially relate to differences in priorities or familiarity with relevant issues between participants. Further training is unlikely to enhance reliability in this case, but raises the question about the importance and specificity of relevant clinical expertise when selecting evaluators. Although our intent was to evaluate the importance of clinical expertise, we classified participants quite broadly. Participants classified as clinical specialists were provided CPGs within their broad area of practice; but, we did not ascertain whether in fact the specific topic of the CPG was an area in which they actually did have knowledge or experience. For example, a clinician with expertise in musculoskeletal practice might practice primarily in a narrower area within the field, such as, rheumatology, upper extremity, lower extremity, joint replacements etc. In such cases, their familiarity with the salient features of the specific conditions might be less detailed and result in a differential evaluation regarding whether the clinical population had been appropriately defined. Future investigations should focus on content knowledge in a more specific sense to determine the importance of this issue when selecting evaluators.

It is not uncommon for the policy developers to use reliability data to set standards for the number of appraisers required when establishing guideline evaluation processes. Our data provide little direction in this regard, except to suggest that more than two appraisers are advisable. This is consistent with the minimum recommendation of 2 made by the AGREE Collaboration. [7] Measurement theory suggest that additional appraisers/ratings should produces higher levels of reliability[20] as do the AGREE Collaboration when stating that 4 appraisers is preferable. Our data did not follow this trend, beyond three appraisers. Our study evaluated the reliability of appraisers performing their assessment in isolation. This process replicates that which might be used by a working committee independently assessing quality scores. However, individual therapists might also need to evaluate CPG when no committee has been established to do so. In these cases, we would recommend three or more appraisers should still be recruited. We recognize that when evaluating CPGs, it is not just the score, but the process that is important. Adding additional appraisers with different perspectives may increase the variability/disagreements. In fact, our data support this, as quadratic weighting of disagreements improved reliability coefficients in generalists and researchers, but not specialists. Specialists tended to disagree on whether a CPG should be used are not, but were less uncertain about that recommendation. We suggest the ideal approach to evaluation of CPGs is one where a group of potential end-users with clinical and guideline expertise work together to review a potential CPG by using the AGREE to facilitate a consensus process to determine both the quality and relevance to practice. These quality ratings should be disseminated to the relevant clinical communities. We recognize that the ideal process will not always be possible. Individual clinicians who must make decisions about utilization of CPGs should recruit colleagues to assist with the evaluation process before modifying their clinical practice based on CPGs with unknown validity.

Although interpretation of validity analyses is complex and requires some subjective decisions, overall our validity analyses are supportive of the AGREE instrument and are substantively similar to that reported by the AGREE Collaboration for 33 medically based CPGs[14] A factor analysis structure which suggests that the concepts measured by the AGREE are similar to the Domains described by the AGREE underlies the content and structural validity of the scale. The factor analysis was strongly supportive of the domains of Stakeholder Involvement and Editorial Independence. This would support uniqueness of items within these domains. The largest factor contained items from the domain Rigor of Development as well as the first two items in Scope and Purpose. Conceptually these two items, which require specific objectives and a well-defined clinical question, fit well with a process of rigorous development. That the largest factor relates to methodological issues supports the AGREE as an evaluative tool. The second factor contained the items regarding whether the patients were specifically described, whether procedures for updating the guideline were provided, all of the items from the applicability domain and 3/4 items from the clarity and presentation. Clarity of presentation, applicability and a defined patient population may all relate to the ability to implement CPGs and thus all retain a conceptual relationship to the domains Applicability or Clarity of Presentation described by the AGREE Collaboration. The third factor contained all items of the stakeholder involvement as well as the additional question in rigor of development addressing whether experts had externally reviewed the guideline. In general, these results support stakeholder involvement as a subscale and include external involvement within this distinct domain. The fourth factor contained only two items, one the item on health benefits/ side effects and a second whether recommendations were specific and unambiguous. This factor explained a small percentage of the variance and contained only two items, making it difficult to relate these results to the factor analysis presented by the AGREE Collaboration. While the results vary somewhat from those reported by the AGREE Collaboration, there is substantial similarity, particularly when one examines the concepts that are represented by the items that clumped together. We view our factor analysis results with caution given the small sample sizes. The AGREE Collaboration study use 100 guidelines and conducted this analysis during the field-testing prior to inclusion of item 23. Our analysis included only 56 guidelines. We did not conduct formal sample size calculations, as practical limitations on guideline availability determined our sample size. Rules of thumb suggest ten "subjects" per item for factor analysis, requiring 230 CPGs for a well-powered analysis. Thus, some inherent instability should be expected in the factor analyses conducted to date. With the proliferation of CPG, we expect that larger samples might be evaluated in future studies and provide more definitive results on the factor validity of the AGREE instrument.

Construct validity tests also supported the validity of the AGREE instrument. Constructs were developed to replicate those used in the previous validation of the AGREE[14] and provided similar findings. Despite our inability to be confident about whether some CPGs had a methodologist, we still found large differences between the Rigour of Development Domain score obtained where we could identify a team containing a methodologist as compared to where this was not present or unclear (21 point difference). The AGREE Collaboration found a 9 point advantage in this domain for guidelines developed within a guideline development program and 19 points for those developed as national initiatives. Similarly, our correlations between overall assessment scores and those reported for Rigor of Development (0.64) concurred with those reported by AGREE Collaboration data (0.87) in that both suggest a substantive relationship.

Overall, our findings were remarkably consistent with those reported by the AGREE collaboration when validating the AGREE as used by medical practitioners[14] Validation by independent groups in different settings provides a stronger foundation for any instrument. These findings can be used to increase confidence in the current practice of different health care disciplines to use the AGREE to facilitate the evaluation of CPGs.

## Conclusion

1. The AGREE instrument is reliable and valid when used by physical therapists to assess the quality of clinical practice guidelines

2. A minimum of three appraisers should be used to optimise reliability, although issues on effecting knowledge transfer or maximizing validity might increase these requirements.

3. There is no evidence that specific types of physical therapists provide more reliable scores. As this study did not address whether familiarity with the actual content of the CPG influenced reliability, this should be studied in the future.

4. There is evidence that guidelines developed with the assistance of experienced guideline developers are more rigorous.

5. The AGREE instrument was found to be useful in assisting both novice and experienced therapists in evaluation of clinical practice guidelines. The majority of therapists would continue to use the instrument.

6. Validity analysis supported the majority of results reported by the AGREE Collaboration)[14] The type of CPGs and evaluators were different in the current study supporting the validity of this instrument across a spectrum of circumstances.

## Competing interests

IG is an associate member of the AGREE Collaboration.

## Authors' contributions

JM, DB, SS, SSW formed the original study team which conceived of the research question, obtained the ethics approval and obtained grant funding. JM developed the research design, conducted statistical analyses, developed and facilitated the training sessions, developed and evaluated the student evaluations and drafted the manuscript. DB managed grant funds, selection of clinical practice guidelines, appraiser recruitment and data collection. All authors approved the final study protocol, contributed to interpretation of the study results and participated in revisions of the manuscript. IG provided consultation with respect to CPG research and health care policy. All authors read and approved the final manuscript.

## Pre-publication history

The pre-publication history for this paper can be accessed here:



## Supplementary Material

Additional File 1 The training test Click here for file
