# Supplementary material for: Reliability and validity of the AGREE instrument used by physical therapists in assessment of clinical practice guidelines
Source: BMC Health Serv Res. 2005 Mar 2;5:18. doi: 10.1186/1472-6963-5-18 (PMC555572; doi:10.1186/1472-6963-5-18)
Supplement: Additional File 1 — The training test [file 1472-6963-5-18-S1.doc]

**Study Self -Test on the AGREE Instrument**

A. The purpose of the AGREE instrument is to

1. Facilitate a consensus on the true quality for a clinical practice guideline

2. Provide a framework for assessing the quality of clinical practice guidelines

3. Provide an indication on the level of evidence for specific treatment interventions

4. Determine whether a given clinical practice guideline has sufficient quality to have a positive impact on patient management.

Answer 2.

B. Which of the following aspects of quality does the AGREE instrument assess?

1. Quality of reporting

2. Quality of the individual studies upon which the recommendations are based

3. Quality of some aspects of the recommendations

4. Both 1 and 3

Answer 4.

C. Which of the following types of guidelines can the AGREE instrument be used to evaluate?

1. New guidelines

2. Existing guidelines

3. Updates of existing guidelines

4. All of the above

Answer 4.

D. Which of the following is NOT a domain captured by the AGREE instrument

1. Impact on outcomes

2. Rigor of development

3. Stakeholder involvement

4. Scope and purpose

Answer 1.

E. The domain on editorial independence evaluates which of the following items

1. Whether the guidelines is editorially independent from the funding body

2. Whether conflicts of interest in development of the guidelines have been recorded

3. Whether steps have been taken to resolve conflicts of interest with stakeholders during development of the guideline

4. Both 1 and 2

answer 4.

F. If you are confident that a criterion for a given item on the AGREE has been fully met, you should answer with which of the following responses?

1. Agree or disagree depending on the extent to which you think the issue has been addressed

2. Strongly agree

3. Agree

4. Either one or three

answer 2.

G. The overall assessment included at the end of the AGREE is to be completed based on which of the following criteria?

1. The total score

2. The extent to which adequate scores are received by the six domains

3. The judgment of the appraiser with respect to the overall quality taking each of the appraisal criteria into account

4. None of the above

answer 3.

H. Which of the following statements is NOT true regarding involvement of relevant professional groups, as evaluated by the AGREE?

1. It includes members of the steering group, or the research team who selected and rated the evidence

2. It includes individuals involved in formulating the final recommendations

3. It includes individuals who externally reviewed the guideline

4. It should contain information about the composition, discipline and relevant experience of the guideline development group

Answer 3.

I. Which of the following is NOT described by the AGREE as a means to indicate that patient preferences have been considered?

1. Involvement of patient representatives

2. Information was obtained from patient interviews

3. Experienced clinicians provided information on patient disabilities

4. Literature reviews of patient experiences were included

answer 3.

J. Which of the following is NOT a guideline item, regarding stakeholder involvement, on the AGREE?

1. The guideline has been piloted among target users

2. The patients to whom the guideline is meant to apply are specifically described

3. The clinical questions covered by the guideline are specifically described

4. The costs of implementation are considered

answer 4.

K. Which of the following is required to indicate that a systematic review was undertaken, as described by the AGREE?

1. The search terms used should be described

2. Resources consulted should be listed and may include electronic databases and databases of systematic reviews

3. The dates of literature searched should be listed

4. All the above

answer 4.

L. Which of the following items does NOT pertain to the Rigor of Development domain of the AGREE?

1. The criteria for selecting the evidence are clearly described

2. The methods used for formulating recommendations are clearly described.

3. The health benefits, side effects and risks have been considered in formulating recommendations.

For. A sensitivity analysis is performed to determine the impact of the excluded studies.

Answer 4.

M. Which of the following items does NOT pertain to the Rigor of Development domain of the AGREE?

1. There is an explicit link between the recommendations and the supporting evidence.

2. The guideline has been externally reviewed by experts prior to its publication.

3. A procedure for public input into the guidelines is provided.

4. A procedure for updating the guideline is provided.

Answer 3.

N. Which of the following items does NOT pertain to the Clarity and Presentation domain of the AGREE

1. Different options for management of the condition are clearly presented.

2. The guideline is supported with statements from expert reviewers.

3. The key recommendations are easily identifiable.

4. The recommendations are specific and unambiguous.

Answer 2.

O. Which of the following are NOT listed by the AGREE, as appropriate tools for application that should accompany the guideline to encourage it’s implementation?

1. A summary document

2. Computer support

3. Educational tools

4. Statements from expert reviewers

answer 4.

P. Which of the following items does NOT pertain to the Applicability domain of the AGREE?

1. Potential societal and cultural barriers to applying the recommendations have been discussed

2. Potential cost implications of applying the recommendations have been considered

3. The potential organizational barriers in applying the recommendations have been discussed

4. The guideline presents key review criteria for monitoring and/or audit purposes

Answer 1.

Developed by Joy MacDermid 2003- based on the AGREE collaboration. Appraisal of guidelines for research and evaluation (AGREE). (www.agreecollaboration.org)
